# When the Eyes Deceive: Uncommon Ophthalmic Presentation of Giant Cell Arteritis

**DOI:** 10.7759/cureus.98164

**Published:** 2025-11-30

**Authors:** Tony Yap, Leena Yalakki-Jagadeesh, Alison Penwarden, Khushbakht Subhani

**Affiliations:** 1 General Medicine, Basingstoke and North Hampshire Hospital, Basingstoke, GBR; 2 Rheumatology, Basingstoke and North Hampshire Hospital, Basingstoke, GBR; 3 Ophthalmology, Basingstoke and North Hampshire Hospital, Basingstoke, GBR

**Keywords:** 4th cranial nerve palsy, anterior ischaemic optic neuropathy, gca, giant cell arteritis (gca), large vessel vasculitis and gca

## Abstract

Giant cell arteritis (GCA) is a systemic granulomatous large vessel vasculitis that predominantly affects older adults. We present a rare case of a patient in her 60s who presented with hypertension and isolated cranial nerve (CN) IV palsy. Initial imaging and temporal artery biopsy performed were negative. Her symptoms then resurfaced again after her prednisolone was stopped. Re-commencement of steroids led to a quick resolution of her visual symptoms and headaches within a matter of days.

This case highlights the importance of clinical judgement and prompt initiation of steroids without delay. A gradual and prolonged steroid tapering regime is also essential to avoid visual loss, the most feared complication in GCA.

## Introduction

Giant cell arteritis (GCA) is a systemic granulomatous vasculitis that predominantly affects older adults [1]. It primarily involves large and medium-sized arteries, particularly the branches of the external carotid artery, such as the temporal artery. Its classical symptoms include headaches, reduced or loss of visual acuity and jaw claudication, corresponding to the supplying arteries that are affected. Whilst diplopia is a specific indicator for GCA with cranial nerve (CN) III involvement, CN IV involvement is rarely described [1]. Visual loss, the most feared complication, usually results from arteritic anterior ischaemic optic neuropathy (AAION), occurring in up to 20% of untreated patients [2].

Systemic symptoms may not always present in GCA, at times only with visual signs and symptoms as described as occult GCA [3]. CN involvement in GCA is rare and typically affects CN III or VI [4,5]. Isolated CN IV palsy has been reported only once in the literature [6]. Temporal artery biopsy remains the gold standard for diagnosis but is subject to false-negatives due to skip lesions [7]. Early diagnosis and treatment are essential, as delayed therapy can result in permanent visual impairment [1,7].

We present a rare case of GCA initially manifesting as isolated CN IV palsy and delayed recurrence manifesting as AAION.

## Case presentation

We present a 67-year-old lady with a background of right knee meniscal tear, shingles, and psoriasis. She is fully independent and physically active, with no personal or family history of autoimmune or early cardiovascular disease. She is a non-smoker.

First presentation

She initially presented with a hypertension of 185/100 mmHg and a one-day history of sudden-onset binocular vertical diplopia, accompanied by right retro-orbital pain and nausea worsened by eye movement. She had to patch her right eye to manage her diplopia. She also described intermittent, dull right orbital and jaw pain, occurring twice weekly for approximately 20 minutes. Similar, milder episodes had occurred four months earlier and had self-resolved. The pain improved with paracetamol, and nausea was exacerbated when looking down.

She denied headache, scalp tenderness, jaw claudication, vision loss, shoulder or hip girdle symptoms, fever, weight loss, or night sweats. There were no signs of dental issues, morning stiffness, photosensitivity, Raynaud’s phenomenon, cough, or alopecia. Joint and systemic examinations were unremarkable. Her investigations are as detailed in Table 1.

**Table 1:** Investigation results from first admission

Investigation	Result	Reference Range
C-Reactive Protein	8 mg/L	0-5 mg/L
Erythrocyte Sedimentation Rate	26 mm/h	0-20mm/h
White Cell Count	6.4x10^9^/L	4-11x10^9^/L
Electrophoresis Immunoglobulin G (IgG)	11.5 g/L	7-16 g/L

Ocular exam revealed a full range of extraocular movement with no ophthalmoplegia. Stroke was the top differential diagnosis then, and she was loaded on 300 mg aspirin.

**Figure 1:**
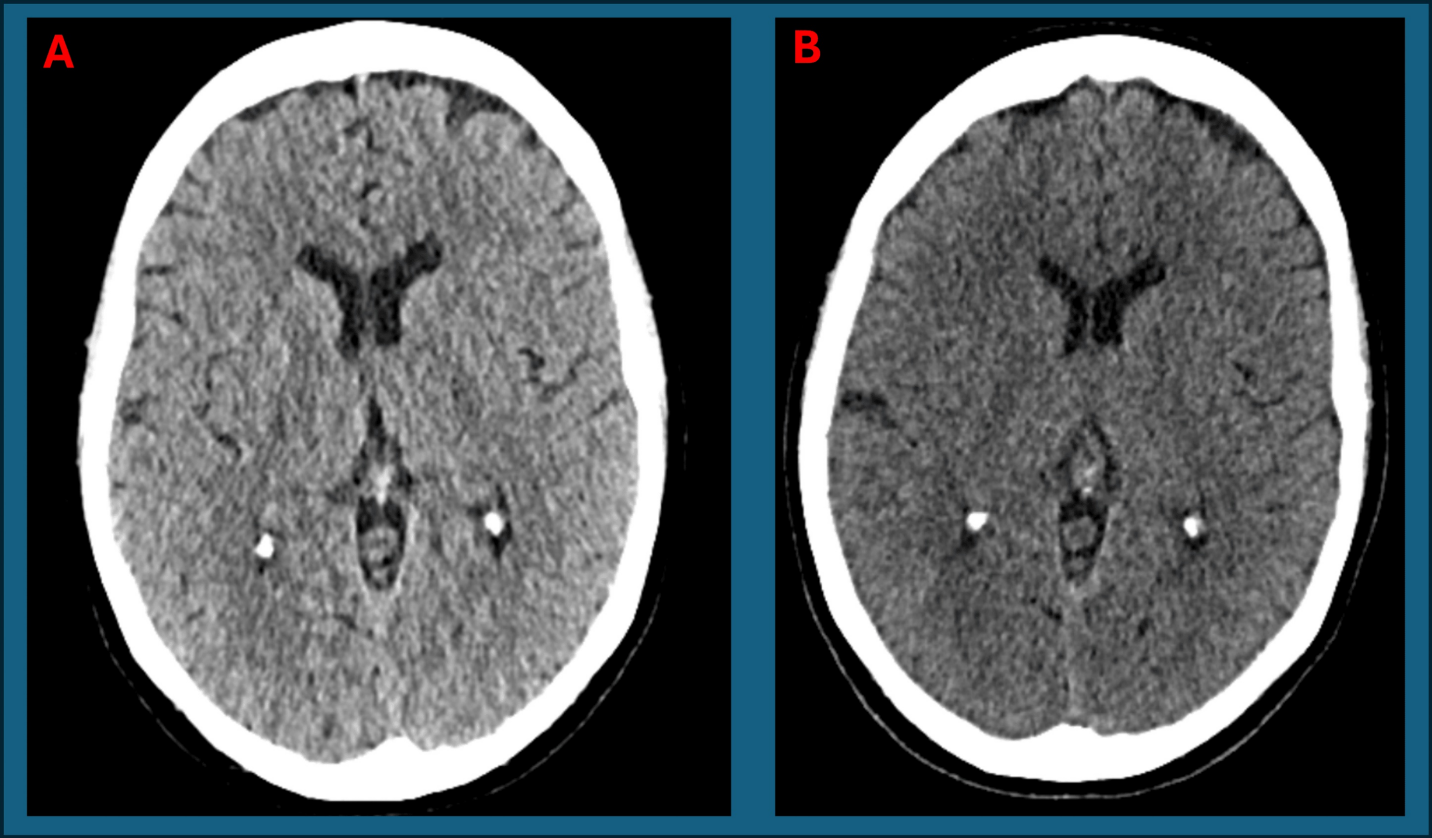
A: Normal axial CT brain from first presentation. B: Normal axial CT brain from second presentation

**Figure 2:**
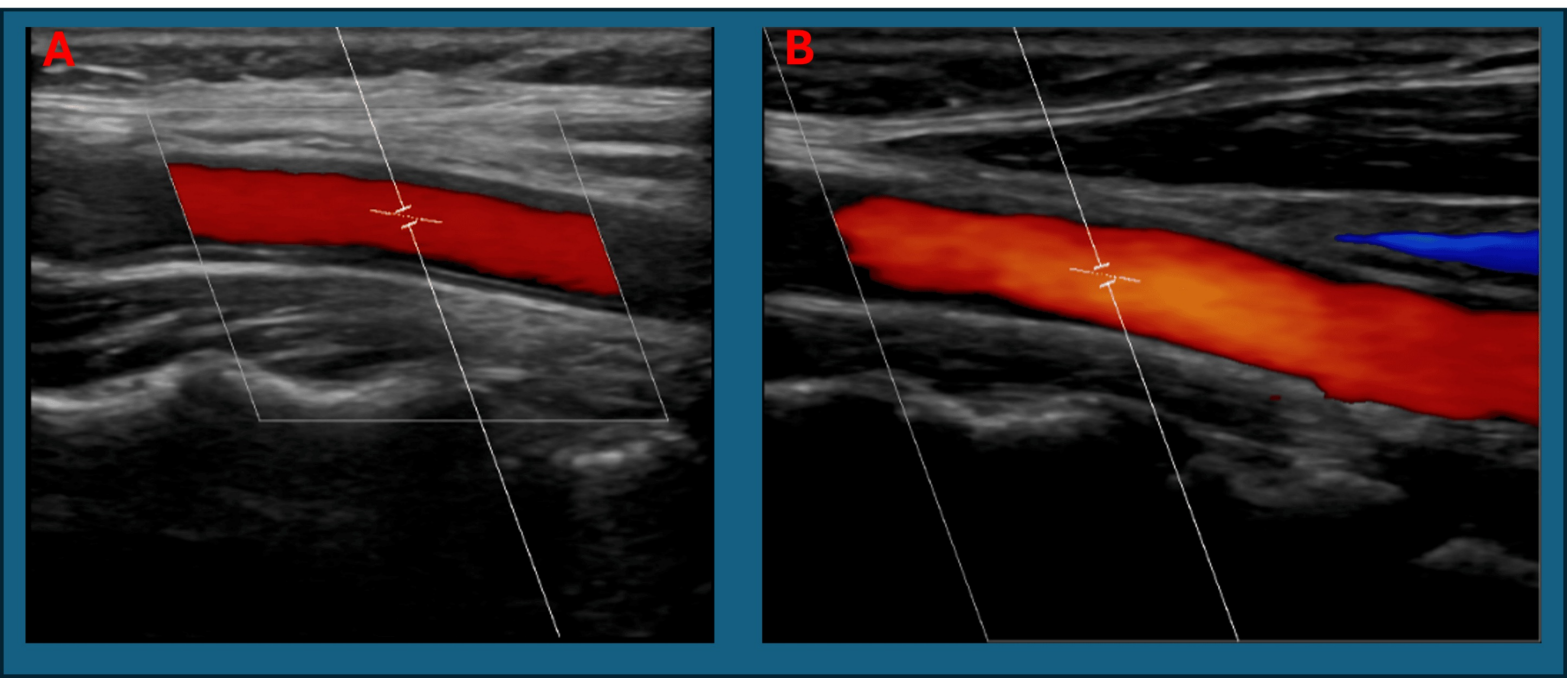
A: Normal ultrasound carotid Doppler of right common carotid artery (peak systolic velocity: 112.2 cm/s, end-diastolic velocity: 22.4 cm/s). B: Normal ultrasound carotid Doppler of left common carotid artery (peak systolic velocity: 129.0 cm/s, end-diastolic velocity: 19.5 cm/s)

**Figure 3:**
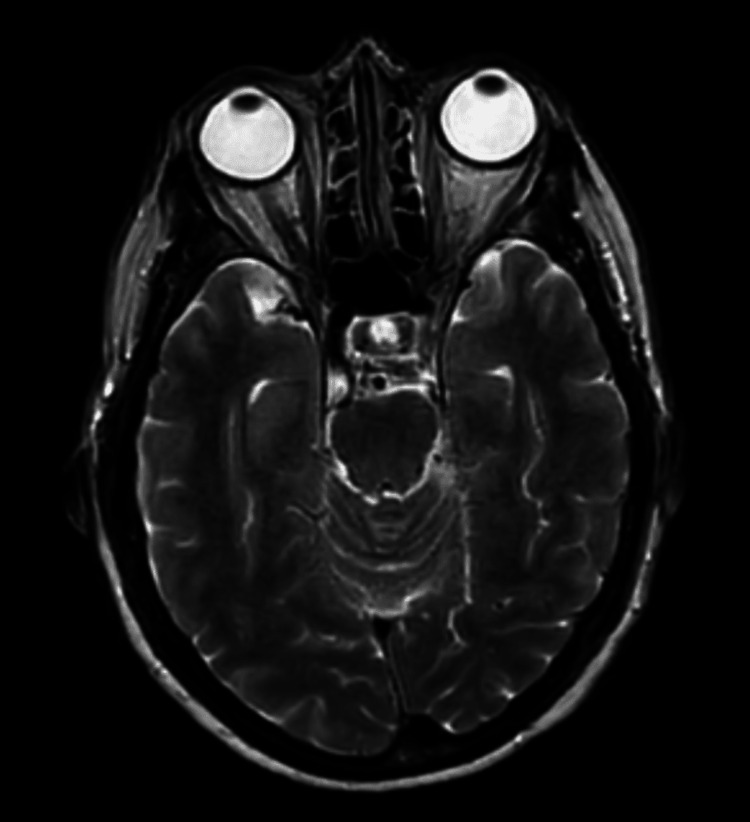
Normal axial T2 MRI head sequence from first presentation

Her CT brain was normal (Figure 1). A carotid Doppler ultrasound was performed and was normal (Figure 2). MRI brain was unremarkable (Figure 3). GCA was still felt to be unlikely then.

Ophthalmology identified a positive Parks-Bielchowsky test, suggestive of isolated right CN IV palsy. Fundoscopic exam showed a healthy optic nerve and fundi and no relative afferent pupillary defect (RAPD) was seen. Pupils were equally reactive. The best corrected visual acuity was 0.08 (right) and 0.06 (left).

Rheumatology assessment on day four of her admission noted non-thickened and pulsatile temporal arteries. She had no proximal weakness. Her GCA probability score was 12 given cranial nerve involvement, headache, duration of symptoms and demographics. This placed her in the intermediate risk category [8]. She was started on 60 mg of prednisolone with a tapering plan and discharged after six days of admission.

On outpatient review, her right CN IV palsy had resolved with no superior oblique muscle weakness and she returned to driving. Right temporal artery biopsy revealed only thickening of the internal elastic lamina, consistent with age-related changes. Steroids were discontinued due to low suspicion of GCA.

Three weeks after stopping steroids, her right-sided facial pain returned. Her general physician (GP) suspected trigeminal neuralgia and started Amitriptyline, which provided partial relief.

Second presentation

However, three months later, she presented with worsening headache described as a tight band, photophobia, blurred right vision, and nocturnal awakening due to pain.

**Figure 4:**
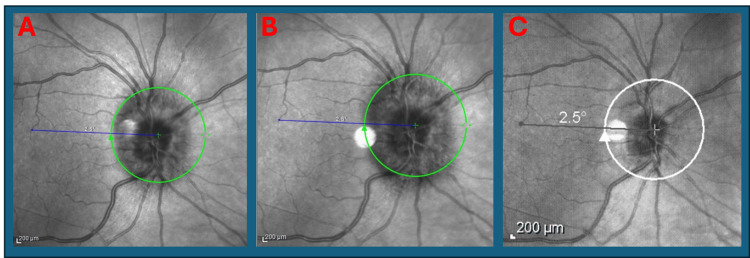
A: Optical coherence tomography (OCT) image of right eye from first presentation in the second recurrence showing swollen optic disc. B: OCT image from ophthalmology follow-up in second recurrence showing persistent right swollen optic disc. C: OCT image three weeks post-discharge from second recurrence showing resolution and normal right optic disc.

**Figure 5:**
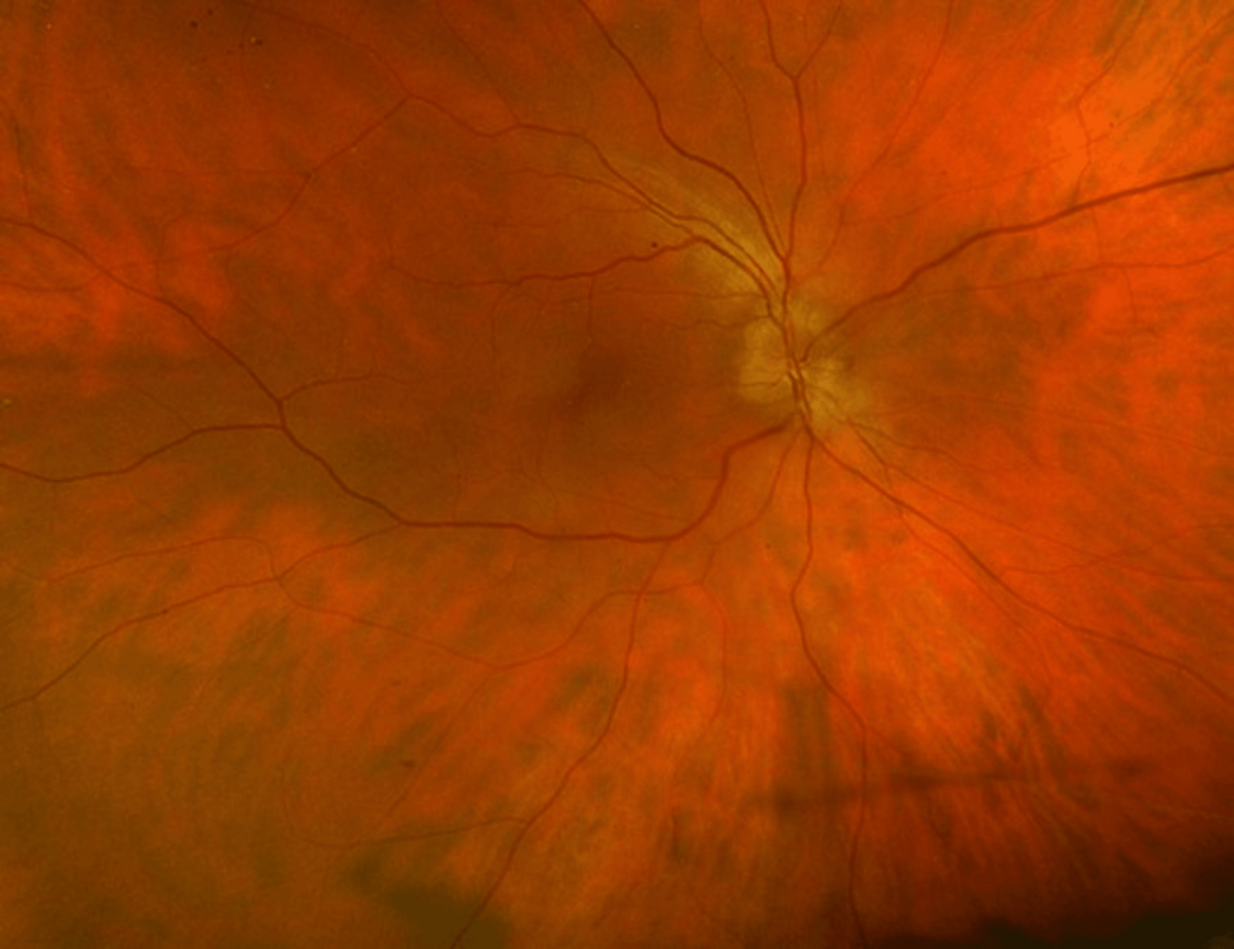
Fundus photograph of the right fundus on re-presentation

On re-evaluation, she had significant right optic disc swelling (Figures 4, 5). Visual acuity was 6/12 (right) and 6/6 (left), with a marked reduction in colour vision (Ishihara: 2/21 right vs 21/21 left). A slight RAPD was present, and visual field testing revealed central and inferior field loss in the right eye. Her extraocular range of motion, retina and macula were normal.

Two weeks later, her right visual acuity deteriorated further to 6/60, whilst left eye was 6/9. Intraocular pressure was 14 mmHg (right) and 6 mmHg (left). The right pupil was sluggish, and optic disc oedema persisted. There was no conjunctival injection. Her blood pressure was elevated at 190/123 mmHg. Her investigation results are as detailed in Table 2.

**Table 2:** Investigations results from the second admission

Investigation	Result	Reference Ranges
C-Reactive protein	11 mg/L	0-5 mg/L
Erythrocyte sedimentation rate	22 mm/h	0-20 mm/h
White cell count	6.1x10^9^/L	4-11x10^9^/L
Ferritin	179 µg/L	30-400 µg/L
Connective tissue disease screen	Negative	Screened for Ro, La, Sm, RNP, Jo-1, Scl-70, dsDNA, Centromere, Mi-2, Ku, Th/To, RNA Polymerase III, PM-Scl, PCNA, Ribosomal P protein
Anti-myeloperoxidase abs	<3.2 CU	0-19 CU
Anti-proteinase 3 abs	<2.3	0-19 CU
Immunoglobulin (IgG) electrophoresis	13.0 g/L	7-16 g/L

She was admitted for treatment with pulsed intravenous methylprednisolone for 1 g per day for three days in view of ocular involvement followed by a tapering course of oral prednisolone from 60 mg. This led to complete resolution in terms of her headache, pain, and colour vision within days.

**Figure 6:**
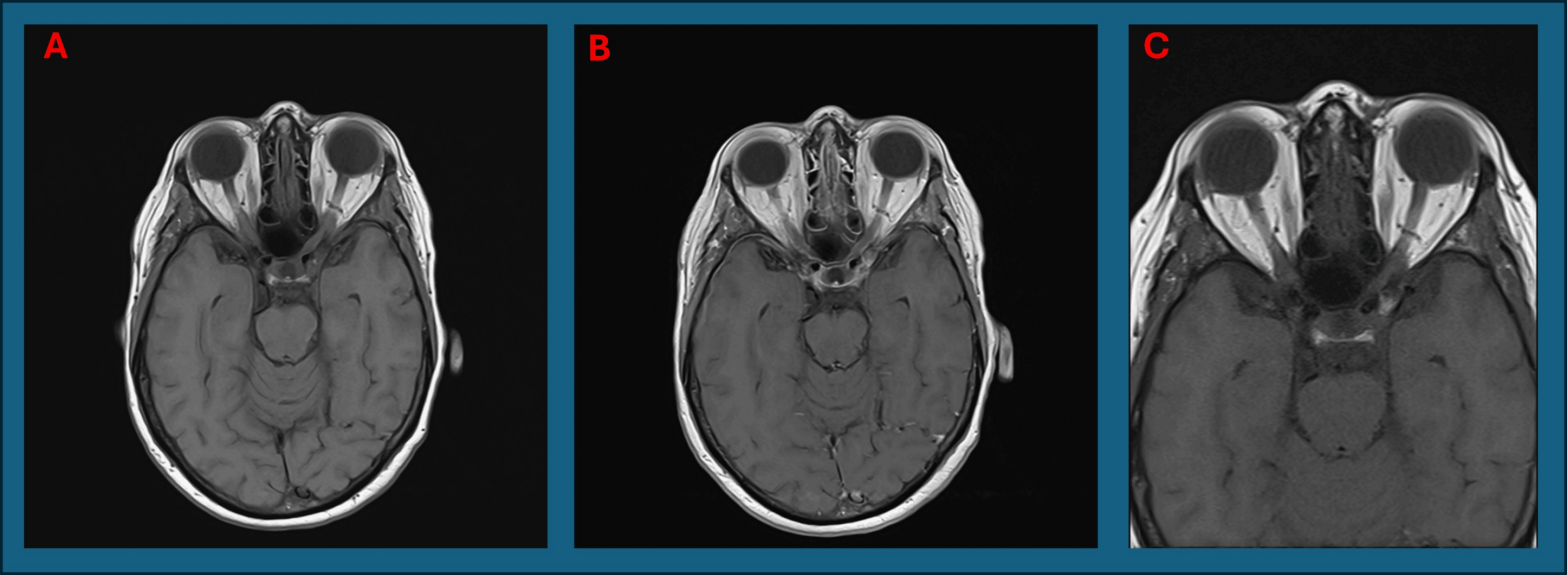
A: Normal axial T1 MRI head sequence pre-contrast from second presentation. B: Normal axial T1 MRI head sequence post-contrast from second presentation. C: Normal Axial T1 MRI Head sequence no contrast used on outpatient review after second discharge

**Figure 7:**
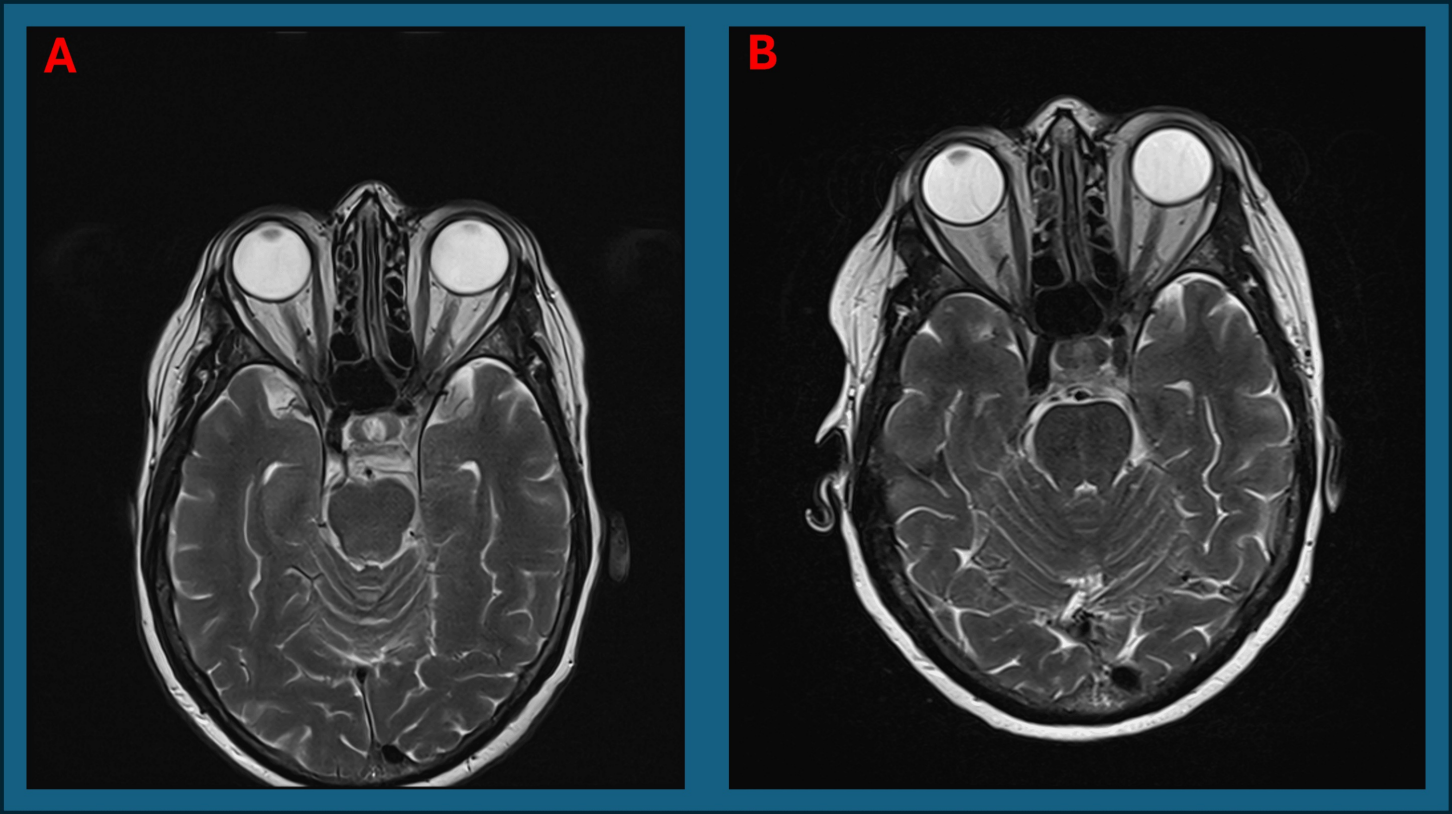
A: Normal axial T2 MRI orbit sequence from second presentation. B: Normal axial T2 MRI orbit sequence on outpatient review after second discharge

**Figure 8:**
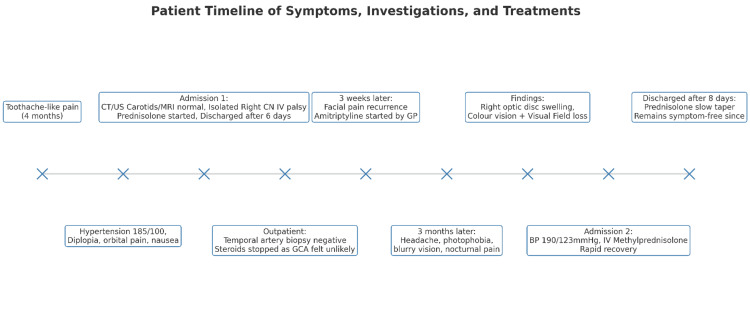
Timeline of symptoms, investigations and treatments

On examination, the right temporal artery was pulseless. MRI of the head and orbits with contrast were normal (Figures 6, 7). Aquaporin-4 antibodies was negative. Despite atypical features, the overall clinical picture was in keeping with AAION secondary to GCA. The patient's symptom identification, investigation, and treatment timeline is given in Figure 8.

The patient was then discharged after eight days of admission with the following the prednisolone tapering regime (Table 3). Safety net advice was also given to seek medical attention and increase her prednisolone dose should her symptoms recur. She was followed up shortly with neurology in the outpatient setting. She was discharged from the neurology service due to reassuring findings. She had an MRI head and orbit as an outpatient, which did not show any intracranial pathology nor cavernous sinus lesion.

**Table 3:** Prednisolone tapering regime

Prednisolone tapering regime	
Dose of Prednisolone	Duration
60 mg daily	1 week
50 mg daily	1 week
40 mg daily	2 weeks
35 mg daily	2 weeks
30 mg daily	2 weeks
25 mg daily	2 weeks
20 mg daily	2 weeks
Thereafter, weaning of 2.5mg every 4 weeks until 10mg	

She was then seen in the rheumatology clinic three weeks later with complete resolution and no recurrence of her symptoms.

She was also reviewed by the ophthalmology team and orthoptist in the outpatient setting again with reassuring eye exam and normal optical coherence tomography (OCT). Her visual acuity returned to 6/6 in both eyes with intraocular pressure of 14 mmHg in the right eye and 16 mmHg in the left eye. Fundus examination showed a healthy optic disc, with no significant visual field defect and colour vision on Ishihara chart was full bilaterally.

## Discussion

The initial presentation of diplopia in GCA is uncommon, observed in only about 5% of cases, most often due to CN VI palsy in isolation followed by CN III palsy [4,5]. In this case, the absence of classical features such as jaw claudication or visual loss, along with a negative temporal artery biopsy, contributed to diagnostic uncertainty. However, temporal artery biopsy has a sensitivity of 85%-90%, and false-negatives due to skip lesions are well documented up to 7% despite being the gold standard investigation [9].

Isolated CN IV palsy in GCA is extremely rare, with only one previously reported case [6]. The reported case had a similar presentation to our encounter with rapid response to prednisolone; however, the patient’s temporal artery biopsy being positive was helpful in clinching the diagnosis [6]. The reported cases of GCA presenting with CN VI palsy were reassuring in terms of response to steroid treatment in more than 90% of patients, which may be mirrored in CN IV palsy presentations too [4].

Furthermore, inflammatory markers such as C-reactive protein (CRP) and erythrocyte sedimentation rate (ESR) may be normal in about 7%-20% of patients with GCA [10]. The patient’s hypertension may have contributed to her cranial ischaemic complications, although hypertension itself is not a known risk factor for developing GCA [11]. Nonetheless, it highlights the importance of recognizing vascular risk factors in GCA management.

The European Alliance of Associations for Rheumatology (EULAR) recommends a slow corticosteroid taper over 12 months or more from a starting dose of 40-60 mg a day [7]. In this case, steroids were tapered over 14 weeks rapidly and discontinued, which may have led to an inadequate treatment response and subsequent relapse. A more gradual and lengthier approach to steroid tapering was adopted for the second presentation and the patient remained symptom-free 4.5 months since the onset.

It has been shown that immediate treatment along with prompt workup within 24 hours of presentation can reduce the risk of permanent visual loss [12]. Furthermore, it has been reported that untreated AAION secondary to GCA may lead to contralateral involvement within 10 days [2]. Therefore, in this case, prompt recognition of atypical GCA features at the second presentation, coupled with timely reinstitution of corticosteroids, resulted in reversal of visual symptoms, underscoring the importance of clinical judgment even in the face of negative biopsy and imaging.

## Conclusions

This case illustrates a rare presentation of GCA with isolated cranial nerve IV palsy, followed by delayed AAION. It highlights the need for heightened clinical suspicion of GCA in elderly patients presenting with unexplained diplopia, even in the absence of classical features or a positive biopsy. Atypical presentations should not delay corticosteroid initiation, and a prolonged tapering regimen is essential to prevent relapse and irreversible visual loss.
